# Targeting the mTOR-Autophagy Axis: Unveiling Therapeutic Potentials in Osteoporosis

**DOI:** 10.3390/biom14111452

**Published:** 2024-11-15

**Authors:** Rongjin Chen, Chenhui Yang, Fei Yang, Ao Yang, Hefang Xiao, Bo Peng, Changshun Chen, Bin Geng, Yayi Xia

**Affiliations:** 1 Department of Orthopedics, The Second Hospital of Lanzhou University, Lanzhou 730030, China; 120220901411@lzu.edu.cn (R.C.); 120220901481@lzu.edu.cn (C.Y.); 120220901491@lzu.edu.cn (F.Y.); yanga21@lzu.edu.cn (A.Y.); 120220901471@lzu.edu.cn (H.X.); pengb20@lzu.edu.cn (B.P.); chencs666@ynu.edu.cn (C.C.); ery_gengb@lzu.edu.cn (B.G.); 2 Orthopedic Clinical Medical Research Center and Intelligent Orthopedic Industry Technology Center of Gansu Province, Lanzhou 730030, China; 3 The Second Clinical Medical School, Lanzhou University, Lanzhou 730030, China; 4 Department of Orthopedics, Tianshui Hand and Foot Surgery Hospital, Tianshui 741000, China

**Keywords:** osteoporosis, mTOR signaling pathway, autophagy, bone metabolism, therapeutic targeting

## Abstract

Osteoporosis (OP) is a widespread age-related disorder marked by decreased bone density and increased fracture risk, presenting a significant public health challenge. Central to the development and progression of OP is the dysregulation of the mechanistic target of the rapamycin (mTOR)-signaling pathway, which plays a critical role in cellular processes including autophagy, growth, and proliferation. The mTOR-autophagy axis is emerging as a promising therapeutic target due to its regulatory capacity in bone metabolism and homeostasis. This review aims to (1) elucidate the role of mTOR signaling in bone metabolism and its dysregulation in OP, (2) explore the interplay between mTOR and autophagy in the context of bone cell activity, and (3) assess the therapeutic potential of targeting the mTOR pathway with modulators as innovative strategies for OP treatment. By examining the interactions among autophagy, mTOR, and OP, including insights from various types of OP and the impact on different bone cells, this review underscores the complexity of mTOR’s role in bone health. Despite advances, significant gaps remain in understanding the detailed mechanisms of mTOR’s effects on autophagy and bone cell function, highlighting the need for comprehensive clinical trials to establish the efficacy and safety of mTOR inhibitors in OP management. Future research directions include clarifying mTOR’s molecular interactions with bone metabolism and investigating the combined benefits of mTOR modulation with other therapeutic approaches. Addressing these challenges is crucial for developing more effective treatments and improving outcomes for individuals with OP, thereby unveiling the therapeutic potentials of targeting the mTOR-autophagy axis in this prevalent disease.

## 1. Introduction

Osteoporosis (OP) is a pervasive skeletal disorder that poses a significant challenge to public health worldwide, especially affecting the elderly and postmenopausal women [1,2]. Characterized by a progressive decline in bone mass and structural integrity, OP increases susceptibility to fractures, thereby leading to a substantial burden on individuals and healthcare systems [3,4]. The pathophysiology of OP is multifaceted, involving complex interactions between genetic, hormonal, and environmental factors. Among the myriad of cellular processes implicated in the regulation of bone homeostasis, autophagy and the mechanistic target of the rapamycin (mTOR) pathway have emerged as critical players [5,6,7]. These processes not only influence bone cell lifespan and function but also modulate the response of bone tissue to various stressors and therapeutic agents.

Autophagy, a cellular self-digestion process, plays a pivotal role in removing damaged organelles and proteins, thus maintaining cellular health and contributing to the dynamic balance between bone formation and resorption [7,8,9]. Dysregulation of autophagy has been implicated in the pathogenesis of various forms of OP, including senile OP, postmenopausal OP, glucocorticoid-induced OP, and diabetic OP. On the other hand, mTOR, a central regulator of cell growth and metabolism, intersects with autophagy by acting as a critical checkpoint [10,11,12], thereby influencing bone density and quality.

The intricate interplay between autophagy, mTOR signaling, and bone metabolism presents a promising avenue for therapeutic intervention. However, despite the potential of targeting these pathways to modulate bone health, the clinical application of such strategies remains underexplored. This review seeks to bridge this gap by providing a comprehensive analysis of the current understanding of the interactions among autophagy, mTOR, and OP. We aim to elucidate the mechanisms by which autophagy and mTOR contribute to bone health, explore the potential of these pathways as therapeutic targets, and assess the efficacy of mTOR modulators in the treatment of OP.

By integrating insights from molecular biology, pharmacology, and clinical research, this review endeavors to highlight the potential of autophagy and mTOR as novel targets for OP therapy. Through a detailed examination of the evidence, we summarize innovative approaches for enhancing bone health and outline future directions for research in this dynamic field. Our goal is to provide a foundation for the development of targeted interventions that could mitigate the impact of OP, improving outcomes for patients worldwide. 

## 2. Exploring the Complex Interplay: Autophagy, mTOR Signaling, and Their Impact on Osteoporosis

### 2.1. Autophagy Mechanisms in Osteoporosis: Insights and Implications

**Figure 1:**
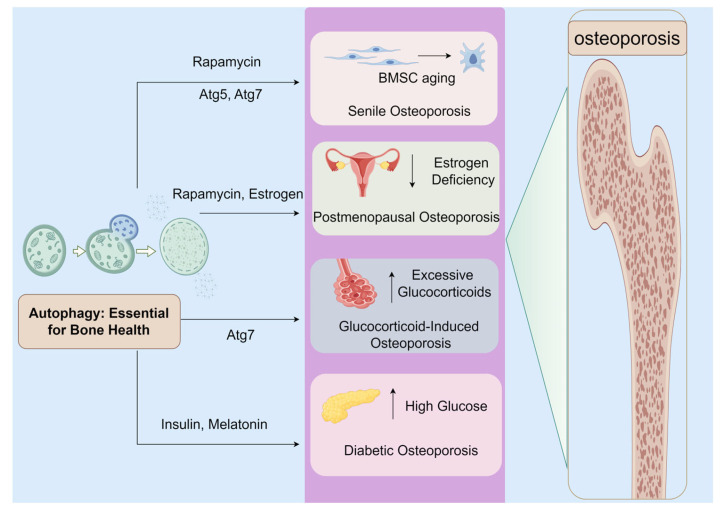
Autophagy Mechanisms in Osteoporosis. Created by Figdraw.com (https://www.figdraw.com).

Autophagy, often referred to as type II programmed cell death, is a crucial cellular process for degrading damaged, misfolded, or dysfunctional proteins and organelles, directing them to lysosomes. This process is vital for maintaining cellular homeostasis, particularly under nutrient deprivation and stress, thus promoting cell survival [13]. However, dysregulation of autophagy can lead to pathological changes and contribute to the development of OP, as depicted in Figure 1.

OP manifests in various forms, each with distinct triggers but connected by a common thread: the disruption of autophagy. Senile osteoporosis, primarily due to aging, leads to a significant reduction in bone mass and deterioration of bone microarchitecture, increasing the risk of fractures. Research indicates that autophagy’s decline in bone marrow stromal cells (BMSC) from OP patients correlates with decreased osteogenic potential, which can be reversed by activating autophagy through treatments like rapamycin [14,15]. This suggests autophagy’s protective role in mitigating bone loss, where specific genetic deletions related to autophagy, such as Atg7 [16] and Atg5 [17], mirror aging effects on bone mass and contribute to age-related low bone mass phenotypes. Conversely, upregulation of autophagy in osteoblasts (OBs) promotes bone formation, highlighting autophagy’s crucial role in maintaining skeletal integrity and suggesting its decline as a primary factor in age-related bone loss [18].

Postmenopausal osteoporosis, a prevalent form of OP, arises from a significant decrease in estrogen levels, disrupting the balance between bone resorption and formation [19]. Autophagy upregulation through rapamycin treatment rescues BMSC functionality and attenuates the postmenopausal OP phenotype [20]. Estrogen deficiency accelerates osteoclastogenesis and suppresses autophagy in bone cells, leading to increased apoptosis [21], whereas estrogen treatment [22] and compounds like resveratrol [23] can boost autophagy, enhancing bone cell viability and restoring balance in bone metabolism in postmenopausal OP rats.

Glucocorticoid (GC)-induced osteoporosis (GIOP) presents another scenario where autophagy plays a mitigating role against GC-induced apoptosis and bone loss [24]. Selective deletion of Atg7 in monocytes suppresses osteoclast (OC) differentiation and reduces bone quality damage in GIOP models [25]. The interaction between GCs and cellular autophagy is dose-dependent, with low doses initiating beneficial autophagy for OB survival, while high doses lead to accelerated apoptosis [26]. This nuanced interaction suggests that autophagy can offer a protective effect on OBs in the presence of GCs, contingent upon the dosage [27].

Diabetic OP disrupts bone homeostasis by affecting both OBs and OCs [28]. BMSCs from diabetic patients exhibit reduced osteogenic differentiation and autophagy levels, yet treatments like insulin can mitigate these effects, promoting bone healing [29]. High glucose levels reduce autophagy during OC formation, but enhancing autophagy can promote OC generation [30], with melatonin modulating autophagy levels in OBs, offering a potential therapeutic avenue for delaying diabetes-induced OP [31]. 

Beyond these conditions, autophagy’s role extends to various other forms of osteoporosis, including drug-induced and disuse osteoporosis. However, research in these areas remains limited, necessitating further investigation to fully comprehend autophagy’s implications across different osteoporosis types. This unified examination underscores the multifaceted role of autophagy in osteoporosis, highlighting its potential as a therapeutic target across the spectrum of the disease.

### 2.2. Relation Between mTOR and Autophagy

#### 2.2.1. Structure of mTOR

mTOR, the target of rapamycin (sirolimus), is a macrolide produced by streptomyces hygroscopius. It forms two distinct complexes: mTOR complex 1 (mTORC1) and mTOR complex 2 (mTORC2), which differ in their sensitivity to rapamycin as well as their upstream and downstream interactions [32]. Both complexes comprise large assemblies with unique and shared components. Specifically, mTORC1 includes the regulatory-associated protein of mTOR (Raptor) and proline-rich AKT substrate 40 kDa (PRAS40), while mTORC2 is characterized by mammalian stress-activated map kinase-interacting protein 1 (mSIN1), rapamycin-insensitive companion of mTOR (Rictor), and proteins Protor 1 and 2. Shared elements in both complexes consist of mTOR itself, mammalian Lethal with Sec-13 protein 8 (mLST8), DEP-domain-containing mTOR-interacting protein (Deptor), and Telomere maintenance 2 (Tel2) along with Tel2 interacting protein 1 (Tti1) [33]. 

Raptor plays a pivotal role in mTORC1, crucial for its stability, localization to lysosomal surfaces, substrate recognition, and overall function [34,35,36,37,38]. In contrast, PRAS40 acts as an intrinsic inhibitor of mTORC1 by binding to Raptor, thus competing with other substrates and inhibiting downstream signaling [39,40,41].

While significant advances have been made in characterizing mTORC1, understanding of mTORC2 is rapidly evolving. mSIN1, a key component of the mTORC2 complex, possesses a N-terminal domain (NTD), a RAS-binding domain (RBD), a conserved region in the middle (CRIM), and a pleckstrin homology (PH) domain at its C-terminus. The RBD and PH domains are critical for mTORC2 activation through their interactions with active RAS and lipid membranes, while the CRIM domain is essential for substrate recruitment [42,43,44]. Additionally, mSIN1 stabilizes the mTORC2 complex by binding Rictor via its NTD, thereby connecting Rictor to mLST8 [44,45]. Similar to Raptor in mTORC1, Rictor regulates mTORC2 assembly, stability, and activity, with its C-terminal domain contributing to the complex’s rapamycin insensitivity [44,46]. Protor, existing in two isoforms, interacts with Rictor via a conserved N-terminal region, though its role is still not fully understood [47,48]. 

**Figure 2:**
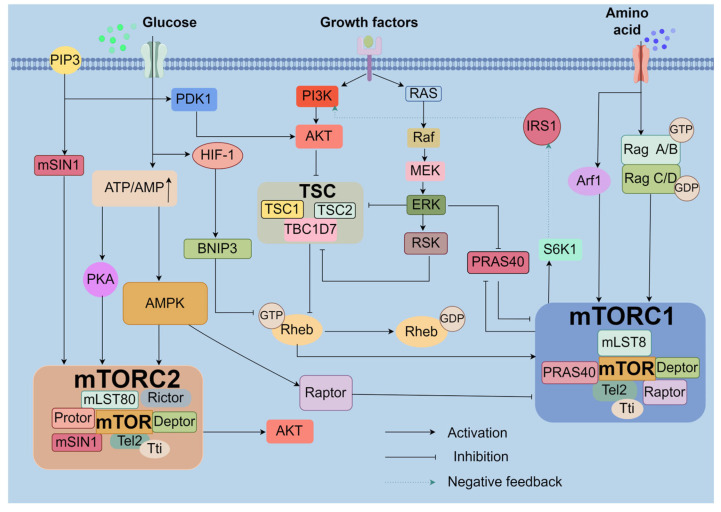
Overview of MTOR signal pathway. mTOR, Mammalian target of rapamycin; mTORC1,Complex 1 of mTOR; mTORC2, Complex 2 of mTOR; Raptor, mTOR-associated regulatory protein; Deptor, mTOR-interacting protein with DEP domain; PRAS40, 40 kDa substrate for AKT rich in proline; mLST8, Sec-13 protein 8 with mammalian lethal function; Rictor, Companion of mTOR insensitive to rapamycin; mSIN1, Protein interacting with stress-activated MAP kinase in mammals; Protor, Protein observed alongside Rictor;Tel2, Maintenance protein 2 for telomeres; Tti1,Protein interacting with Tel2; PI3K, Phosphatidylinositol-3-kinase enzyme; PIP3, Phosphatidylinositol-3,4,5-triphosphate; PDK1, Kinase 1 dependent on phosphoinositides; AKT, Protein kinase B; MEK, Protein kinase activated by mitogens; ERK, Kinase regulated by extracellular signals; TSC, Sclerosis tuberous complex; GAP, Protein that activates GTPase; Arf1, ADP-ribosylation factor 1; AMPK, Kinase activated by adenosine 5’-monophosphate; HIF-1, Hypoxia-inducible factor 1; S6K1, Kinase 1 of ribosomal S6; PKA, Protein kinase activated by cAMP. Created by Figdraw.com (https://www.figdraw.com).

Among the shared components, mLST8 is particularly critical for mTORC2, as its knockdown disrupts mTORC2 substrate activation without affecting mTORC1 substrate phosphorylation [49]. This specificity is due to mLST8’s interaction with mTORC2 cofactors Rictor and mSIN1, which enhances complex assembly [50]. Furthermore, Tel2 and Tti1, which constitutively interact with mTOR in both complexes, are vital for maintaining the integrity of mTORC1 and mTORC2; their knockdown results in the disassembly of both complexes [51]. Lastly, Deptor, a conserved protein that binds to mTOR via its PDZ domain, inhibits the activity of both mTORC1 and mTORC2 [52]. However, mTOR kinase activity phosphorylates Deptor, promoting its release and thereby reversing its inhibitory effects (Figure 2).

#### 2.2.2. Regulation of the mTOR Signal Pathway

Belonging to the phosphoinositide-3-OH kinase (PI3K)-related kinase family, mTOR, an atypical serine/threonine protein kinase, plays a pivotal role in regulating cellular growth and metabolism in response to nutritional and hormonal signals [53]. This regulation is achieved through the activation of mTOR Complex 1 (mTORC1) by several growth factors that interact with cell-surface receptor tyrosine kinases, thereby initiating the PI3K/AKT and RAS/ERK (extracellular-signal-regulated kinase) pathways [54,55]. AKT and ERK further amplify mTORC1 activity by inhibiting its negative regulators, notably the TSC complex and PRAS40 [56,57,58]. The TSC complex, comprising TSC1, TSC2, and TBC1D7, keeps Rheb in a dormant state through its GAP (GTPase-activating protein) activity and Rheb ubiquitination [59,60]. Upon growth factor stimulation, AKT-mediated phosphorylation of TSC2 and USP4 (ubiquitin-specific peptidase 4) liberates Rheb from TSC inhibition, while PRAS40, regulated by AKT and mTORC1, modulates mTORC1 activity through phosphorylation at specific residues [61,62,63].

Amino acid availability also plays a crucial role in mTORC1 activation by affecting its subcellular localization, with Rag GTPases being central to this process [64,65]. Active Rags, in the presence of abundant amino acids, interact with v-ATPase-Regulator to recruit mTORC1 to lysosomal membranes where Rheb resides, thereby facilitating mTORC1 activation [66,67]. Interestingly, glutamine and asparagine activate mTORC1 in a Rag-independent manner through ADP-ribosylation factor 1 (Arf1), highlighting the complexity of mTORC1 regulation and the need for further exploration [68].

Energy stress triggers mTORC1 inhibition predominantly via an adenosine 5’-monophosphate-activated protein kinase (AMPK)-dependent pathway [58,69]. Decreased ATP levels during glucose deprivation lead to AMPK activation, which in turn inhibits mTORC1 through the activation of the TSC complex and direct phosphorylation of mTOR and Raptor [70,71,72,73]. Additionally, mTORC1 can be regulated by AMPK-independent pathways under stress conditions, such as hypoxia, where HIF-1 inhibits mTORC1 through BNIP3 induction or DDIT4/REDD1 activation [74,75].

In contrast, the regulation of mTOR Complex 2 (mTORC2) is more enigmatic and complex. It is believed that mTORC2 activation relies on PI3K/PIP3 signaling, where growth factor stimulation leads to the recruitment of PDK1 and AKT to the membrane, facilitating mTORC2 activation and subsequent full AKT activation through phosphorylation [76,77]. mTORC2 is also responsive to adrenergic signaling and can be positively regulated by AMPK independently of mTORC1 [78,79]. However, mTORC1 can negatively regulate mTORC2, illustrating the intricate feedback mechanisms governing mTOR signaling and its pivotal role in cellular physiology [80]. Finally, mTORC2 activity is negatively regulated by mTORC1, as elevated mTORC1 activity from insulin/IGF-1 signaling increases S6K1 activity, which phosphorylates insulin receptor substrate 1 (IRS1) on negative regulatory sites, inhibiting PI3K signaling and thus reducing mTORC2 activity. This intricate interplay between mTORC1 and mTORC2, and their regulation by various stimuli, underscores the potential of targeting mTOR-mediated autophagy as a promising strategy for OP treatment, offering new avenues for therapeutic intervention (Figure 2). 

#### 2.2.3. mTOR: The Driving Force of Autophagy Dynamics

Over recent decades, significant strides have been made in understanding the role of mTOR as a pivotal inhibitor of autophagy, exerting its influence across transcriptional, translational, and post-translational modifications. At the autophagy induction phase, mTORC1 suppresses the activity of the ULK1 complex by phosphorylating key components ULK1 and ATG13, thereby inhibiting the initiation of autophagy [81]. Intriguingly, under conditions of nutrient deprivation, the inactivation of mTORC1 leads to the dephosphorylation and activation of the ULK1 complex, facilitating autophagy induction. Notably, the inhibition of mTOR also activates DAP1, establishing a sophisticated regulatory network designed to balance autophagic activity and prevent cell death due to excessive autophagy [82].

Furthermore, a feedback regulation mechanism between the ULK1 complex and mTORC1 has been identified. Overexpression of ULK1 directly suppresses mTORC1 kinase activity, promoting comprehensive autophagy induction [83]. However, excessive autophagy can trigger cell death, suggesting that ULK1 also activates mTORC1 by inhibiting AMPK, providing a potential negative feedback loop to prevent overactive autophagy [84].

mTOR also plays a critical role in the initiation and nucleation of autophagosomes. It regulates the process by phosphorylating AMBRA1, thus inhibiting the activation of the VPS34 complex, a key event in autophagy initiation [85]. In the later stages of autophagy, mTOR reactivates and induces autophagic lysosome reformation (ALR), maintaining lysosomal homeostasis and establishing a negative feedback loop to avoid excessive autophagy [86].

Lastly, mTOR is involved in the transcriptional regulation of autophagy-related genes by phosphorylating transcription factors EB (TFEB) and E3 (TFE3), thereby restricting the expression of genes crucial for autophagy, further fine-tuning the autophagic process [87,88]. These insights reveal the multifaceted and complex mechanisms by which mTOR regulates autophagy, providing important molecular targets for the development of novel therapeutic strategies (Figure 2).

### 2.3. Interactions Among Autophagy, mTOR, and Osteoporosis

The intricate interplay between autophagy, the mechanistic target of rapamycin (mTOR) pathway, and OP forms a complex regulatory network with profound implications for bone health. The mTOR pathway, a pivotal regulator of cellular growth and metabolism, intricately modulates autophagy, thereby directly influencing various aspects of bone biology. Notably, the mTORC1 complex, by inhibiting the autophagy-related kinase ULK1 and negatively regulating AMP-activated protein kinase (AMPK), significantly impacts autophagic flux [89]. This regulatory mechanism is crucial for maintaining cellular homeostasis and plays a key role in the pathophysiology of OP, a condition characterized by reduced bone mass and an increased risk of fractures.

However, autophagy, as a key pathway for maintaining cellular and physiological functions through the recycling of damaged proteins and organelles, plays a pivotal role in bone metabolism. Its importance is particularly evident in supporting the functions of osteoblasts (OBs) and osteoclasts (OCs), the primary participants in bone formation and resorption, respectively [90,91]. The involvement of autophagy in the differentiation process of OCs, as well as its role in supporting the differentiation of OB precursors, underscores its significance in maintaining bone health [90,91]. Moreover, the FAK family kinase 200 kDa interacting protein (FIP200) gene is crucial for initiating autophagy. Its deficiency compromises bone formation, as evidenced by knockout experiments revealing defects in OB autophagy, including elevated p62 expression, inadequate LC3-II conversion, and reduced GFP-LC3 expression—all leading to impaired bone formation capabilities [7]. 

The dual role of mTOR, capable of both exacerbating and alleviating OP under different conditions, reveals the complexity of its function in bone health. By affecting upstream signals that control autophagy, mTOR directly engages in the activities of BMSCs and OBs in bone formation, as well as in OC-mediated bone resorption [92,93,94]. Understanding how mTOR modulates the progression of OP through the regulation of autophagy opens new avenues for developing therapeutic strategies against this widespread condition.

Moreover, mTOR’s role as a critical sensor of cellular nutritional status and its significant impact on autophagy regulation further emphasize the complex interactions among signaling pathways [95]. Research demonstrating the modulation of autophagy through the AMPK-mTOR axis highlights the importance of regulating autophagy for maintaining cellular and physiological balance [96,97]. This dynamic regulatory mechanism, involving the inhibition of autophagy under nutrient-rich conditions through the phosphorylation of the ULK1 complex by mTOR, and promoting autophagy under nutrient-poor conditions, showcases the pathway’s versatility in maintaining cellular and physiological equilibrium [98].

In summary, the interactions among autophagy, mTOR, and OP not only reveal a complex biological regulatory network but also provide promising directions for new therapeutic approaches to OP. As our understanding of these interactions deepens, the potential for developing innovative treatments targeting OP becomes increasingly tangible, offering hope to those affected by this condition.

## 3. Deciphering the Complex Role of mTOR-Mediated Autophagy in Bone Homeostasis

The exploration into the complex role of mTOR-mediated autophagy in bone homeostasis unveils the pivotal position of the mechanistic Target of Rapamycin (mTOR) in maintaining skeletal health. mTOR regulates key cell types involved in bone metabolism, such as OBs, OCs, osteocytes, and BMSCs, influencing both bone formation and resorption processes, thereby ensuring a delicate balance within the bone microenvironment.

### 3.1. mTOR-Mediated Autophagy in Osteoblasts: Regulatory Mechanisms and Implications for Bone Homeostasis and Osteoporosis

Under normal physiological conditions, autophagy is crucial for the survival of OBs. In vitro studies have shown that autophagy can mitigate oxidative stress-induced OB apoptosis [99]. Research indicates that the mTOR signaling pathway not only regulates OB differentiation but also plays a significant role in the proliferation, apoptosis, autophagy, energy metabolism, and multifunctionality of OBs and OB-like cells [92,100]. Chen et al. [101] demonstrated that the bone synthesis factor Wnt7b promotes OB differentiation by activating mTORC1 via the PI3K-AKT pathway. Conversely, targeting and inhibiting mTORC1 signaling obstructs Wnt7b-induced OB differentiation in ST2 cells. Similarly, Xian et al. [102] found that insulin-like growth factor IGF-1 enhances BMSCs OB differentiation through mTOR signaling, thereby maintaining appropriate bone microstructure and density. Recent studies have highlighted that the mTOR/Raptor-S6K1 axis regulates Runx2 expression through estrogen receptor α to promote OB differentiation [103], suggesting that S6K1 is a key downstream regulator of mTORC1 in OBs and revealing how mTOR/Raptor regulates Runx2 expression to enhance bone formation.

**Figure 3:**
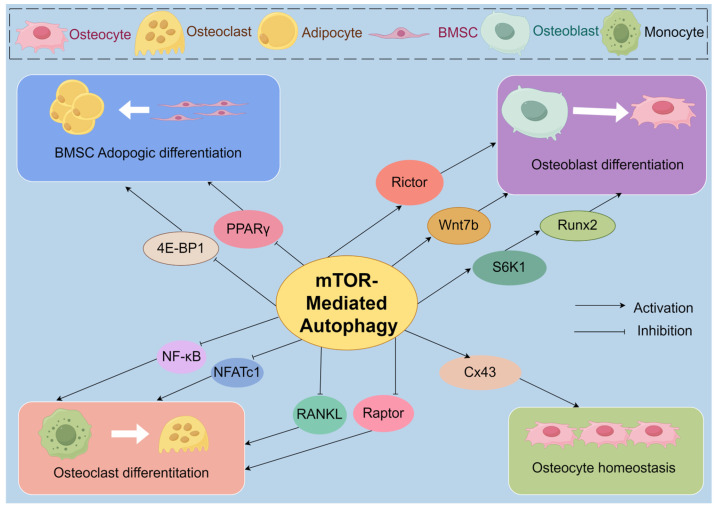
The regulatory role of mTOR-Mediated autophagy in bone homeostasis. PPARγ, Peroxisome proliferator-activated receptor-γ; 4E-BP1, Eukaryotic translation initiation factor 4E (eIF4E)-binding protein 1; NF-κB, Nuclear factor kappa-B; NFATc1, Nuclear factor of activated T cells, cytoplasmic, calcineurin dependent 1; Cx43, Connexin 43; Wnt7b, Wnt Family Member 7B; S6K1, Kinase 1 of ribosomal S6; Runx2, RUNX Family Transcription Factor 2; Rictor, Companion of mTOR insensitive to rapamycin; Raptor, mTOR-associated regulatory protein; RANKL, Receptor Activator of Nuclear Factor-κB Ligand. Created by Figdraw.com (https://www.figdraw.com).

These findings collectively indicate that mTOR acts as a common effector downstream of various bone synthesis metabolic signals and plays a crucial regulatory role in OB proliferation and differentiation through autophagy. However, despite increasing research focusing on the role of mTOR signaling in OB autophagy and its potential involvement in OP pathogenesis, the mechanisms by which upstream signaling molecules activate mTOR pathways and the specific functions of mTORC1 and mTORC2 in bone homeostasis remain unclear. Further studies are needed to elucidate the biological regulatory mechanisms of mTOR in bone homeostasis (Figure 3).

### 3.2. Regulation of Osteoclast Differentiation and Function by mTOR-Mediated Autophagy: Implications for Bone Metabolism and Osteoporosis

Bone resorption is primarily carried out by multinucleated OCs, which originate from hematopoietic progenitor cells and differentiate from monocytes [104]. Research into the role of the mTOR signaling pathway in OC differentiation lags behind that of OB differentiation, leading to some debate regarding its precise function in OC biology. Raptor, a core component of mTORC1, has been implicated in bone metabolism. Zhang et al. [105] observed that mice with a specific knockout of Raptor exhibited reduced bone mass and increased bone fractures. Furthermore, OCs derived from the bone marrow macrophages of these mice showed a marked increase in number. At the cellular and molecular level, mTORC1 has been found to negatively regulate the activation of key transcription factors involved in osteoclastogenesis, namely NF-κB and NFATc1. NF-κB and NFATc1 are crucial for OC formation, indicating that mTORC1 may promote bone formation under normal conditions, while its absence leads to increased OC activation. Rictor, a core component of mTORC2, also plays a role in bone metabolism. Liu et al. [106] demonstrated that Rictor knockout in OB precursors impairs their differentiation into OBs. Additionally, Sun et al. [107] found that RANKL expression was significantly downregulated in Rictor-deficient BMSCs, although OPG and M-CSF levels remained unchanged. This suggests that mTORC2 indirectly regulates OC differentiation through modulation of RANKL expression in BMSCs.

Recent studies [108] have revealed that activation of mTOR can inhibit the differentiation, fusion, and resorptive activity of OCs, while promoting the proliferation of monocytes and mature OCs. Dysregulated autophagy can disrupt OC function, enhancing bone resorption and contributing to OP [109]. Excessive glucocorticoid (GC) use, leading to GIOP, is linked to increased OC autophagy via the PI3K/Akt/mTOR pathway [110]. This overactive autophagy promotes bone resorption and GIOP development. Aoki et al. [111] demonstrated that autophagy significantly increases during OC differentiation and maturation, correlating positively with OC activity and survival. Autophagy inhibitors like chloroquine, 3-MA, and LY294002 can effectively counteract excessive OC activity induced by GCs. Additionally, selective deletion of Atg5 or Atg7 reduces GC-induced OC overactivation and bone loss, preventing OP [25,112]. Atg5 and Atg7 are essential for lysosomal secretion at the OC ruffled border, crucial for bone resorption [113].

These findings suggest that mTOR exerts both direct and indirect regulatory effects on OC differentiation. However, the precise mechanisms by which mTOR regulates OC differentiation and proliferation warrant further investigation (Figure 3).

### 3.3. mTOR-Mediated Autophagy in Bone Marrow Mesenchymal Stem Cells: A Key Regulator of Osteogenesis and Adipogenesis in Bone Health and Disease

BMSCs are undifferentiated cells with the ability to differentiate into OBs, chondrocytes, adipocytes, neurons, and myocytes [114]. Various factors can lead to BMSC aging and apoptosis. Furthermore, autophagy also supports osteogenic differentiation through the PI3K/Akt/mTOR pathway [115]. Research has shown that the AMPK pathway regulates autophagy by early suppression of mTOR and Akt/mTOR, thus modulating MSC osteogenic differentiation [116]. Therefore, autophagy is crucial for maintaining BMSC function and enhancing osteogenic differentiation through the mTOR pathway, highlighting its potential in addressing BMSC-related disorders and improving bone health.

Moreover, bone homeostasis involves a complex interplay of factors. Excessive production of bone marrow adipocytes is a significant risk factor for skeletal health [117], indicating that OP may be related to increased adipose tissue in the bone marrow [118]. Studies have shown that adipogenic factors inhibit osteogenesis, while osteogenic factors suppress adipogenesis [119]. Thus, targeting the inhibition of marrow adipogenesis while promoting osteogenesis could be a promising approach for treating OP.

Inhibition of mTOR signaling has been shown to reduce the adipogenic differentiation of BMSCs. For example, risedronate, a nitrogen-containing bisphosphonate, inhibits the phosphorylation of S6 at S235/236, a direct downstream target of mTORC1, in adipogenically differentiated human mesenchymal stem cells (hMSCs) [120,121].

The nuclear receptor peroxisome proliferator-activated receptor-γ (PPARγ) is a key transcription factor that inhibits osteoblastic differentiation in BMSCs indirectly through an adipogenesis-dependent mechanism, highlighting the interplay between osteoblastic and adipogenic differentiation. Interestingly, mTORC1 plays a key role in PPARγ-mediated fat formation [122]. When PPARγ is overexpressed in OBs, there is a significant reduction in bone mass and an increase in adipogenesis. Furthermore, in vivo studies confirm that PPARγ overexpression in OBs negatively regulates bone mass in male mice and accelerates estrogen-deficiency-related bone loss in females [123]. Additionally, Sun et al. [124] observed increased trabecular bone number in mice with targeted PPARγ knockout and found that endogenous PPARγ in MSCs and OBs inhibits the activity of mTOR downstream factor S6K, reducing osteogenic differentiation. This suggests that PPARγ regulates osteogenesis both directly through the mTOR pathway and indirectly through the regulation of adipogenesis. Given the crucial role of mTOR signaling in bone remodeling, developing mTOR-targeted therapies is of significant importance for treating OP and OP-related fractures (Figure 3).

### 3.4. mTOR-Mediated Autophagy in Osteocytes: Implications for Bone Remodeling and Homeostasis

Osteocytes, which are terminally developed OBs implanted in the mineralized bone matrix, are recognized for their ability to coordinate bone remodeling through the regulation of OB and OC activities, mostly through paracrine processes [125]. They are essential for bone mineral homeostasis, local bone remodeling within their lacunae, and systemic phosphate regulation [126]. To perform these functions, osteocytes synthesize and secrete various regulatory proteins, including SOST, RANKL, dentin matrix acidic phosphoprotein, matrix extracellular phosphoglycoprotein (MEPE), and fibroblast growth factor 23 (FGF23), which are crucial for calcium and phosphorus metabolism [127,128,129,130]. 

Research on mTOR signaling’s role in osteocyte homeostasis is limited. However, recent studies indicate that mTOR signaling may modulate intercellular communication among osteocytes. Connexin 43 (Cx43), a key hemichannel protein, is vital for maintaining dendritic connections between neighboring osteocytes and supporting bone homeostasis both in vivo and in vitro [131,132,133]. Activation of AKT-mTORC1 signaling by dexamethasone has been shown to degrade Cx43, disrupting dendritic processes and impairing cell communication among osteocytes [134]. Similarly, 1,25-dihydroxyvitamin D3 (VitD) promotes autophagy through the Akt/mTOR pathway under hypoxic conditions, thereby preventing osteocyte loss [135]. 

Additionally, recent findings suggest that rapamycin may mitigate age-related trabecular bone changes in older male rats, likely by enhancing osteocyte autophagy. This indicates that mTOR signaling could negatively affect osteocyte autophagy, making rapamycin a potential therapeutic option for senile OP [136]. Therefore, while mTOR signaling appears to adversely impact osteocyte homeostasis, further research is needed to clarify its relationship with osteocyte function (Figure 3).

## 4. Exploring the Role of mTOR Pathway Modulation in Osteoporosis: Therapeutic Insights from Small Molecule Compounds, Clinical Drugs, and Traditional Chinese Medicine

The mTOR signaling pathway is crucial in maintaining bone homeostasis, and its dysregulation is a key factor in OP. Recent research has highlighted the potential of targeting mTOR pathway modulators—both activators and inhibitors—as therapeutic strategies for OP. This section delves into the influence of various factors, including small molecule compounds, clinical drug use, and traditional Chinese medicine, on the mTOR pathway in OP. By elucidating these interactions, we can uncover new approaches for treating OP, underscoring the pathway’s significant role in bone health and disease management.

### 4.1. Small Molecule Compounds

The role of small molecule compounds in the modulation of OB and OC activity has gained significant attention, particularly in the context of OP. These compounds often exert their effects by influencing key signaling pathways and promoting autophagy, which can mitigate the adverse effects of glucocorticoid-induced OP and other forms of bone loss. Cladrin [137] has been shown to downregulate OB activity, leading to enhanced autophagy and reduced apoptosis. Specifically, it activates p-AMPK and increases the ratio of LC3-II to LC3-I, while decreasing p-mTOR levels, Bax, and increasing Bcl-2. These mechanisms underscore its potential in combating glucocorticoid-induced OP. Similarly, Resveratrol [138] downregulates OB function, promoting autophagy through the activation of SIRT1 and increasing LC3-II and Beclin-1 levels, while concurrently inhibiting p-AKT and p-mTOR. This compound’s ability to stimulate autophagy positions it as a promising candidate for therapeutic intervention in glucocorticoid-induced OP. Another notable compound, Geniposide [139], reduces OB activity by downregulating p-PI3K and p-AKT, which leads to an increase in LC3-II and Beclin-1. This compound promotes autophagy and inhibits apoptosis, thereby offering protective effects against glucocorticoid-induced OP. β-Ecdysterone [140] operates through a similar mechanism, decreasing p-mTOR and enhancing autophagy markers such as LC3-II, while also modulating apoptotic proteins like Bax and Bcl-2. This dual action not only promotes cell survival but also supports bone health in glucocorticoid-induced OP. Naringin [141] continues this trend by downregulating key signaling pathways, including p-PI3K and p-AKT, thereby enhancing autophagy as evidenced by elevated Beclin-1 levels. This positions Naringin as another promising agent for improving OB function in the context of OP. Timosaponin BII [142], targeting diabetic OP, has a similar profile, promoting autophagy and inhibiting apoptosis through multiple pathways, including a reduction in p-mTOR and NF-κB activity. Hormonal compounds such as Estradiol [143] and 17beta-estradiol [144] also exhibit downregulatory effects on OBs, promoting autophagy while inhibiting apoptosis through the modulation of p-mTOR and various apoptotic markers. These findings highlight their significance in managing postmenopausal OP. In contrast, Melatonin [145] upregulates OB activity but inhibits autophagy, demonstrating a complex role in microgravity-induced OP. This counterintuitive effect warrants further exploration to fully understand its therapeutic potential. The actions of Monotropein [146] further reinforce the promotion of autophagy in OBs, enhancing cellular resilience against oxidative stress and other detrimental factors associated with OP. Similarly, morroniside [147] also acts on OBs, leading to decreased p-mTOR and increased levels of autophagy-related proteins, including ATG13 and LC3-II, highlighting its beneficial role in postmenopausal OP.

Conversely, certain compounds, such as acacetin [148], have been reported to upregulate signaling pathways that inhibit autophagy in OCs by decreasing p-PI3K, p-AKT, and p-mTOR levels. This inhibition is critical in the context of postmenopausal OP, as it disrupts the balance of bone remodeling. Orcinol glucoside [149] and hydrogen sulfide (H2S) [150] similarly upregulate pathways that ultimately inhibit autophagy in OCs, suggesting a complex interplay between these compounds and cellular signaling pathways that regulate bone health.

On the other hand, various bioactive compounds targeting BMSCs, such as alpinetin [93] and tetramethylpyrazine [151], have been shown to promote autophagy while also enhancing differentiation markers like Runx2 and OCN. This dual action indicates a promising strategy for treating glucocorticoid-induced OP. Additionally, sinomenine [152] and leonurine [115] have demonstrated similar effects, promoting both autophagy and differentiation through modulation of critical signaling pathways.

Interestingly, environmental factors such as bisphenol A [153] and cadmium [154] have adverse effects on osteocytes, leading to downregulation of mTOR and subsequent promotion of autophagy, as well as increased oxidative stress. These findings underline the potential risks associated with certain environmental exposures in the context of OP. Lastly, pinocembrin [155] presents a noteworthy case, as it not only promotes autophagy but also inhibits apoptosis, suggesting a protective role in glucocorticoid-induced OP.

In summary, the intricate interplay of these small molecule compounds highlights their potential as therapeutic agents for combating OP by modulating autophagy and apoptosis in OBs and osteocytes. Future studies should continue to explore these pathways to unlock new treatment strategies for OP and related conditions (Table 1).

### 4.2. Clinical Drug Use

The role of autophagy in OC and OB function is crucial in understanding the mechanisms underlying OP, particularly in the context of various pharmacological interventions. For instance, dexamethasone [110], a commonly used glucocorticoid, has been shown to significantly promote autophagy in OCs. Specifically, it leads to downregulation of key signaling pathways, including p-PI3K, p-AKT, and p-mTOR, while simultaneously increasing levels of LC3-II, Beclin1, Atg1, Atg13, and Atg7. This suggests a complex interplay where glucocorticoids may induce autophagy in OCs, which is particularly relevant for glucocorticoid-induced OP.

In contrast to the aforementioned factors, metformin [156] has emerged as a promising agent for enhancing autophagy in OBs. It achieves this by increasing AMPK activity and decreasing mTOR signaling, which in turn elevates autophagic markers like LC3-II and reduces apoptosis-related proteins. Thus, metformin presents a dual benefit in managing glucocorticoid-induced OP by promoting autophagy while inhibiting cell death.

Additionally, glucosamine [157,158] has been shown to promote autophagy in OBs by downregulating both mTOR and AKT pathways, enhancing autophagic activity as evidenced by increased LC3-II and Beclin1 levels. This reinforces glucosamine’s potential as a therapeutic agent for OP.

In summary, the modulation of autophagy through various clinical drugs and metabolic conditions highlights the intricate balance of signaling pathways that govern bone health. Understanding these interactions not only sheds light on the pathogenesis of OP but also opens avenues for targeted therapeutic strategies that could improve patient outcomes in bone-related disorders. (Table 1)

### 4.3. Traditional Chinese Medicine

Traditional Chinese medicine (TCM) has garnered attention for its potential in managing OP, particularly through the use of herbal combinations like Epimedii Folium and Ligustri Lucidi Fructus [159], which contain the key components Icariin and Salidroside, respectively. Icariin, a major pharmacological component of Epimedii Folium, protects osteocytes from dexamethasone-induced damage and enhances autophagy in bone marrow cells [160,161]. Similarly, Salidroside, the main active ingredient of Ligustri Lucidi Fructus, reduces osteoblast apoptosis under oxidative stress [162]. These herbs have demonstrated the ability to downregulate key signaling pathways, specifically reducing p-mTOR levels while enhancing autophagy markers such as LC3-II and Beclin1. This results in a favorable shift in apoptosis regulation, as indicated by decreased Bax and p53 levels and increased Bcl2 expression, ultimately promoting autophagy and inhibiting apoptosis in OBs. This mechanism shows promise for addressing senile OP.

Conversely, QiangGuYin [163] exhibits a different profile in its action on OBs, where it upregulates p-AKT and p-mTOR signaling, thereby enhancing cell differentiation. However, this comes at the cost of inhibiting autophagy, as evidenced by the reduction in LC3-II/LC3-I ratios and increased levels of p62. Additionally, QiangGuYin appears to decrease RANKL while increasing OPG and RUNX2, further promoting bone formation. This complex interplay underscores the nuanced role of TCM in postmenopausal OP, suggesting that while some formulations may enhance autophagy, others prioritize differentiation and cell survival. Ultimately, these insights into TCM’s mechanisms provide a compelling narrative for its application in OP therapy, highlighting the need for further research to optimize these herbal interventions (Table 1).

### 4.4. The Crucial Role of the mTOR Pathway: From Osteoporosis Management to Advancements in Cancer Therapy

The mTOR pathway is pivotal in controlling cell survival, metabolism, growth, and protein synthesis, bridging both healthy and diseased states, particularly in the realm of cancer. Disruptions in mTOR signaling, often due to genetic mutations at the level of signal transduction, are prevalent across a spectrum of cancers. These disruptions lead to the hyperactivation of mTOR signaling, fueling tumor development and progression. In response, a diverse array of mTOR inhibitors has been crafted and is currently under clinical investigation, with several already securing approval for cancer treatment [164]. Beyond its significance in OP, the mTOR pathway’s role is profoundly critical in bone metastases and the wider sphere of cancer, underscoring the urgent need for in-depth research to unlock the full therapeutic promise of mTOR modulation. Translational research findings, highlighting the mTOR axis’s effectiveness in cancer treatment, advocate for the development of precise preclinical models [165]. These models are essential for a better comprehension of bone cancer dynamics and drug interactions, broadening the utility of mTOR pathway modulation from osteoporosis treatment to promising cancer therapy avenues. This emphasizes the mTOR pathway’s integral function in maintaining bone health and managing disease, and the continuous investigative efforts necessary to discover novel therapeutic approaches.

**Table 1:** Medications Modulating the mTOR Signaling Pathway in Various Types of Osteoporosis.

Names	Status of Use	Cells	mTOR Activity	Mechanisms (↑ Means Increase, ↓ Decrease)	Functions	Promoting or Inhibiting Osteoporosis Type	References
**Small molecule compounds**							
Cladrin	Preclinical	osteoblasts	Downregulated	Cladrin → p-AMPK ↑, p-mTOR ↓, LC3-II/LC3-I ↑, Bax ↓, Bcl2 ↑	Promote autophagy, inhibited apoptosis	Inhibiting Glucocorticoid-induced Osteoporosis	[137]
Resveratrol	Preclinical	osteoblasts	Downregulated	Resveratrol → p-AKT ↓, p-mTOR ↓, SIRT1↑, LC3-II ↑, Beclin-1 ↑	Promote autophagy	Inhibiting Glucocorticoid-induced Osteoporosis	[138]
Geniposide	Preclinical	osteoblasts	Downregulated	Geniposide → p-PI3K ↓, p-AKT ↓, p-mTOR/mTOR ↓, LC3-II/LC3-I ↑, Beclin1 ↑, p62 ↓, caspase-3 ↓	Promote autophagy, inhibited apoptosis	Inhibiting Glucocorticoid-induced Osteoporosis	[139]
β-ecdysterone	Preclinical	osteoblasts	Downregulated	β-ecdysterone → p-mTOR ↓, LC3-II ↑, Bax/Bcl2 ↓, Beclin1 ↑, ATG5 ↑	Promote autophagy, inhibited apoptosis	Inhibiting Glucocorticoid-induced Osteoporosis	[140]
Naringin	Preclinical	osteoblasts	Downregulated	Naringin → p-PI3K ↓, p-AKT ↓, p-mTOR ↓, Beclin1 ↑, p62 ↓	Promote autophagy	Inhibiting Glucocorticoid-induced Osteoporosis	[141]
Timosaponin BII	Preclinical	osteoblasts	Downregulated	Timosaponin BII → p-mTOR ↓, NF-κB ↓, IκB ↓, LC3-II ↑, Beclin1 ↑, Bax ↓, Bcl2 ↑	Promote autophagy, inhibited apoptosis	Inhibiting Diabetic Osteoporosis	[142]
Estradiol	Preclinical	osteoblasts	Downregulated	Estradiol → ERK-1/2 ↑, p-mTOR/mTOR ↓, LC3-II/LC3-I ↑, Beclin1 ↑, Bax/Bcl-2 ↓, caspase-3 ↓	Promote autophagy, inhibited apoptosis	Inhibiting Postmenopausal Osteoporosis	[143]
17beta-estradiol	Preclinical	osteoblasts	Downregulated	17beta-estradiol → p-AMPK ↑, p-mTOR ↓, SIRT1 ↑, LC3II ↑, Beclin-1 ↑, Bcl-2 ↑, caspase-3 ↓	Promote autophagy, inhibited apoptosis	Inhibiting Postmenopausal Osteoporosis	[144]
Melatonin	Preclinical	osteoblasts	Upregulated	Melatonin → p-ERK ↑, p-Akt ↑, p-mTOR/mTOR ↑, LC3-II/LC3-I ↓, Bcl-2 ↑, Bax ↓	Inhibited autophagy	Promoting Microgravity- Osteoporosis	[145]
Monotropein	Preclinical	osteoblasts	Downregulated	Monotropein → p-AKT ↓, p-mTOR ↓, LC3-II/LC3-I ↑, Beclin1 ↑, p70S6K ↓, 4EBP1 ↓	Promote autophagy inhibited Oxidative stress	Inhibiting Osteoporosis	[146]
Morroniside	Preclinical	osteoblasts	Downregulated	Morroniside → p-mTOR ↓, ATG13 ↑, LC3-II/LC3-I ↑, Beclin1 ↑	Promote autophagy	Inhibiting Postmenopausal Osteoporosis	[147]
Acacetin	Preclinical	osteoclasts	Upregulated	Acacetin → p-PI3K ↓, p-AKT ↓, p-mTOR ↓, LC3-II/LC3-I ↑	Inhibited autophagy	Inhibiting Postmenopausal Osteoporosis	[148]
Orcinol glucoside	Preclinical	osteoclasts	Upregulated	Orcinol glucoside → p-mTOR ↑, p-p70S6K ↑, LC3II ↓, Beclin1 ↓, Atg5 ↓, Atg7 ↓, P62 ↑	Inhibited autophagy	Promoting Senile Osteoporosis	[149]
Hydrogen sulphide (H_2_S)	Preclinical	osteoclasts	Upregulated	H_2_S → p-PI3K ↓, p-AKT ↓, p-mTOR ↓, LC3-II/LC3-I ↑, Beclin1 ↑, p62 ↓	Inhibited autophagy	Promoting Osteoporosis	[150]
Alpinetin	Preclinical	BMSCs	Downregulated	Alpinetin → p-PKA ↑, mTOR ↓, Beclin1 ↑, LC3II/LC3I ↑, p62 ↓, Runx2 ↑, OCN ↑, OPN ↑	Promote autophagy, differentiation	Inhibiting Glucocorticoid-induced Osteoporosis	[93]
Tetramethylpyrazine	Preclinical	BMSCs	Downregulated	Tetramethylpyrazine → p-AMPK ↑, p-mTOR ↓, LC3II/LC3I ↑, P62 ↓	Promote autophagy	Inhibiting Glucocorticoid-induced Osteoporosis	[151]
Sinomenine	Preclinical	BMSCs	Downregulated	Sinomenine → p-PI3K ↓, p-AKT ↓, p-mTOR/mTOR ↓, ALP ↓, RUNX2 ↑, COL1A1 ↑	Promote autophagy, differentiation	Inhibiting Postmenopausal Osteoporosis	[152]
Leonurine	Preclinical	BMSCs	Downregulated	Leonurine → p-PI3K ↓, p-AKT ↓, p-mTOR/mTOR ↓, Atg5 ↑, Atg7 ↑, LC3II ↑	Promote autophagy, differentiation	Inhibiting Osteoporosis	[115]
Bisphenol A	Preclinical	osteocytes	Downregulated	Bisphenol A → ROS ↑, p-mTOR/mTOR ↓, p-ULK1/ULK1 ↑, Beclin1 ↑, LC3II ↑, p62 ↓	Promote autophagy, apoptosis	Inhibiting Osteoporosis	[153]
Cadmium	Preclinical	osteocytes	Downregulated	Cadmium → p-PI3K ↓, p-AKT ↓, p-mTOR ↓, LC3II ↑, ATG5 ↑, p62 ↓	Promote autophagy	Inhibiting Osteoporosis	[154]
Pinocembrin	Preclinical	osteocytes	Downregulated	Pinocembrin → p-PI3K ↓, p-AKT ↓, p-mTOR ↓, Beclin1 ↑, LC3II ↑, p62 ↓	Promote autophagy, Inhibited apoptosis	Inhibiting Glucocorticoid-induced Osteoporosis	[155]
**Clinical drug use**							
Dexamethasone	FDA-approved	osteoclasts	Downregulated	Dexamethasone → p-PI3K ↓, p-AKT ↓, p-mTOR ↓, LC3-II/LC3-I ↑, Beclin1 ↑, Atg1 ↑, Atg13 ↑, Atg7 ↑	Promote autophagy	Inhibiting Glucocorticoid-induced Osteoporosis	[110]
Metformin	FDA-approved	osteoblasts	Downregulated	Metformin → p-AMPK/AMPK ↑, p-mTOR/mTOR ↓, p-p70S6K/p70S6K ↓, LC3-II/LC3-I ↑, p62 ↓, caspase-3 ↓, caspase-9 ↓	Promote autophagy, inhibited apoptosis	Inhibiting Glucocorticoid-induced Osteoporosis	[156]
Glucosamine	FDA-approved	osteoblasts	Downregulated	Glucosamine → p-mTOR/mTOR ↓, LC3-II/LC3-I ↑, Beclin1 ↑	Promote autophagy	Inhibiting Osteoporosis	[157]
Glucosamine	FDA-approved	osteoblasts	Downregulated	Glucosamine → p-AKT ↓, p-mTOR ↓, LC3-II/LC3-I ↑, Beclin1 ↑	Promote autophagy	Inhibiting Osteoporosis	[158]
**Traditional Chinese medicine**							
Epimedii Folium + Ligustri Lucidi Fructus	Preclinical	osteoblasts	Downregulated	Epimedii Folium + Ligustri Lucidi Fructus → p-mTOR ↓, LC3-II/LC3-I ↑, Beclin1 ↑, Bax ↓, Bcl2 ↑, p53 ↓	Promote autophagy, inhibited apoptosis	Inhibiting Senile Posteoporosis	[159]
QiangGuYin	Preclinical	osteoblasts	Upregulated	QiangGuYin → p-AKT/AKT ↑, p-mTOR/mTOR ↑, CKIP-1 ↓, LC3II/I ↓, RANKL ↓, p62 ↑, RUNX2 ↑, OPG ↑	Inhibited autophagy, promote differentiation	Inhibiting Postmenopausal Osteoporosis	[163]

## 5. Gene and Non-Coding RNA Regulation of mTOR Activity and Autophagy in Osteoporosis: Mechanisms and Therapeutic Implications

Recent research has highlighted the complex roles of various regulators in modulating mTOR activity and autophagy, which are pivotal in different forms of OP. This part explores how specific genes and non-coding RNAs affect these pathways and their implications for OP management. 

PMAIP1 [94], when upregulated in OBs, significantly impacts mTOR activity, leading to an inhibition of autophagy in postmenopausal OP. This effect is mediated through a reduction in p-AMPK and Beclin1 levels. On the other hand, Meg3 [166] also upregulates mTOR in OBs but contributes to the inhibition of autophagy in diabetic OP. This mechanism involves an intricate interplay of pathways, including PI3K, Akt, and NF-κB, which collectively enhance mTOR activity and affect autophagy.

In contrast, CNR2 [167] uniquely influences bone health by downregulating mTOR in OBs. This process enhances autophagy, as demonstrated by elevated levels of Beclin1 and LC3-II, both of which are essential in addressing general OP. On the other hand, Opg [168] acts through the suppression of OC activity. This suppression triggers a series of molecular changes, including reduced levels of phosphorylated PI3K (p-PI3K), phosphorylated AKT (p-AKT), and phosphorylated mTOR (p-mTOR), coupled with an increase in phosphorylated ULK1 (pULK1), the LC3-II/LC3-I ratio, and Beclin1, as well as a decrease in p62. Such downregulation fosters autophagy, underscored by the heightened levels of Beclin1 and the LC3-II/LC3-I ratio, pivotal in fighting OP. These modifications underscore a complex array of regulatory mechanisms pivotal for bone health.

Similarly, in osteocytes, miR-199a-3p [169] is observed to be downregulated, leading to decreased mTOR activity and subsequent promotion of autophagy in postmenopausal OP. This regulatory shift results in increased levels of LC3-II, further supporting autophagy as a beneficial response. In addition, in glucocorticoid-induced OP, upregulation of miR-let-7f in BMSCs leads to increased levels of mTOR, Beclin-1, ATG12, ATG5, and LC3-II, ultimately inhibiting autophagy [170].

These insights collectively underscore the diverse mechanisms through which different genes, non-coding RNAs, and traditional treatments influence mTOR activity and autophagy, revealing potential therapeutic targets for managing various types of OP (Table 2).

## 6. Conclusions and Future Directions

The exploration of the mTOR-autophagy axis within the context of OP has unveiled a complex yet fascinating landscape of therapeutic potential. As evidenced by the comprehensive analysis presented in this review, the intricate interplay between mTOR signaling, autophagy, and bone metabolism not only deepens our understanding of OP pathophysiology but also opens new avenues for innovative treatment strategies. The regulatory impact of genes, non-coding RNAs, and traditional treatments on mTOR activity and autophagy underscores the multifaceted nature of OP, suggesting that a one-size-fits-all approach may not be sufficient for effective management of this condition.

Looking ahead, several key areas merit further investigation to fully harness the therapeutic potentials of targeting the mTOR-autophagy axis in OP. Firstly, the development of more selective mTOR modulators could provide a means to finely tune autophagy levels, potentially minimizing side effects and enhancing therapeutic outcomes. Secondly, elucidating the detailed molecular mechanisms by which mTOR and autophagy influence bone cell function and bone metabolism is crucial. This knowledge could facilitate the identification of novel biomarkers for early diagnosis and the stratification of patients most likely to benefit from targeted therapies.

Furthermore, comprehensive clinical trials are imperative to establish the efficacy, safety, and optimal dosing regimens of mTOR inhibitors and other agents that modulate autophagy in the context of OP. These studies should aim to include diverse populations to ensure broad applicability of findings. Additionally, the synergistic effects of combining mTOR modulation with other therapeutic approaches, such as hormonal therapy, bone anabolic agents, or lifestyle interventions (e.g., diet and exercise), warrant thorough exploration. Such combination therapies could potentially offer superior outcomes by addressing multiple aspects of OP pathophysiology simultaneously.

In conclusion, the journey to unravel the therapeutic potential of the mTOR-autophagy axis in osteoporosis is only beginning. The promise shown by early studies ignites hope for the development of more effective, targeted treatments that could significantly improve the quality of life for individuals suffering from this debilitating condition. By continuing to push the boundaries of our knowledge and by fostering multidisciplinary collaborations, we stand on the cusp of transforming osteoporosis management. The future holds great promise for turning the tide against osteoporosis, moving from merely slowing disease progression to actively restoring bone health and resilience.

## Figures and Tables

**Table 2:** Regulatory Impact of Genes and Non-coding RNAs on mTOR Activity in Various Types of Osteoporosis.

Names	Cells	mTOR Activity	Mechanisms (↑Means Increase, ↓ Decrease)	Functions	Promoting or Inhibiting Osteoporosis Type	References
**Gene**						
PMAIP1	osteoblasts	Upregulated	PMAIP1 → p-AMPK ↓, p-mTOR ↑, LC3-II ↓, Beclin1 ↓	Inhibited autophagy	Promoting Postmenopausal Osteoporosis	[94]
Meg3	osteoblasts	Upregulated	Meg3 → PI3K ↑, Akt ↑, mTOR ↑, p62 ↑, NF-κB↑	Inhibited autophagy	Promoting Diabetic Osteoporosis	[166]
CNR2	osteoblasts	Downregulated	CNR2 → mTOR ↓, Beclin1 ↑, LC3-II ↑, p62 ↓	Promote autophagy	Inhibiting Osteoporosis	[167]
Opg	osteoclasts	Downregulated	Opg → p-PI3K ↓, p-AKT ↓, p-mTOR ↓, pULK1 ↑, LC3-II/LC3-I ↑, Beclin1 ↑, p62 ↓	Promote autophagy	Inhibiting Osteoporosis	[168]
**No-coding RNA**						
miR-199a-3p	osteocytes	Downregulated	miR-199a-3p → p-mTOR ↓, p-IGF-1 ↓, LC3-II ↑	Promote autophagy	Inhibiting Postmenopausal Osteoporosis	[169]
miR-let-7f	BMSC	Upregulated	miR-let-7f → mTOR ↑, Beclin-1 ↑, ATG12 ↑, ATG5 ↑, LC3-II ↑	Inhibited autophagy	Promoting Glucocorticoid-induced Osteoporosis	[170]

## Data Availability

Not applicable.
